# Pedagogical Proposal of Tele-Exercise Based on “Square Stepping Exercise” in Preschoolers: Study Protocol

**DOI:** 10.3390/ijerph18168649

**Published:** 2021-08-16

**Authors:** Alberto Domínguez-Muñoz, Jorge Carlos-Vivas, Sabina Barrios-Fernandez, José Carmelo Adsuar, Jesús Morenas-Martín, Miguel Angel Garcia-Gordillo, Francisco Javier Domínguez-Muñoz

**Affiliations:** 1 Faculty of Sport Science, University of Extremadura, 10003 Cáceres, Spain; alberto.dominguez@elejerciciotecuida.com; 2 Promoting a Healthy Society Research Group (PHeSO), Faculty of Sport Sciences, University of Extremadura, 10003 Cáceres, Spain; jadssal@unex.es; 3 Social Impact and Innovation in Health (InHEALTH) Research Group, Faculty of Sport Sciences, University of Extremadura, 10003 Cáceres, Spain; 4 Motor Control Research Group, Faculty of Sport Sciences, University of Extremadura, 10003 Cáceres, Spain; jesusmorenas@unex.es; 5 Universidad Autónoma de Chile, Sede Talca 3467987, Chile; miguel.garcia@uautonoma.cl; 6 Physical Activity and Quality of Life Research Group (AFYCAV), Faculty of Sport Sciences, University of Extremadura, 10003 Cáceres, Spain; fjdominguez@unex.es

**Keywords:** tele-square-stepping exercise, early education, psychomotor skills, balance, cognitive skills, pedagogy

## Abstract

Early childhood education aims to achieve the motor, cognitive, emotional, and social development of preschoolers by providing them with a variety of learning opportunities. The square-stepping exercise (SSE) is a balance and lower limb strength training programme used to prevent falls and stimulate cognitive function in older adults. This project aims to propose an SSE tele-exercise (Tele-SSE) protocol to evaluate its effects on the motor and cognitive development of children aged between 3 and 6 years. A randomized controlled trial with experimental (Tele-SSE) and control (general education) groups will be carried out. The application of Tele-SSE will be performed for 9 months (three times per week) and one additional follow-up after the intervention at the beginning of the next academic year. One-hundred and two preschoolers will be recruited and randomly distributed into the two groups: experimental (*n* = 51) and control (*n* = 51). Although the main outcome will be balance due to the nature of the SSE, outcomes will include physical and motor (body mass index, waist circumference, handgrip and lower-limb strength, speed-agility, and cardiorespiratory fitness) and cognitive (executive functions and attention, episodic memory, and language assessment, using the Fitness Assessment in the Preschool Battery (PREFIT) and The National Institutes of Health Toolbox—Early Childhood Cognition Battery. This project aims to improve cognitive and motor skills in preschoolers aged between 3 and 6 years old, based on a 9-month Tele-SSE intervention. If this intervention proves to be effective, it could be implemented in those centres, entities and associations specializing in early childhood education.

## 1. Introduction

Enabling children to achieve their full developmental potential is one of the main objectives to be covered by teachers through educational action. The Spanish Early Childhood Education Curriculum states that all educational actions will aim to contribute to the physical, affective, social, and intellectual development of children. Furthermore, this document adds that attention must be paid to achieve properly affective development, movement and body control habits, communication and language, coexistence of elementary patterns and social relations, as well as to the discovery of environmental physical and social characteristics, focusing on sensory education [1].

Likewise, this document also indicates that the second cycle of childhood education (from 3 to 5−6 years old) will contribute to the development of children’s capacities that will allow them to know their own and other children’s bodies, acquire coordination and general dynamic control, act safely, and learn to value and respect personal differences. Thus, the development of activities that encourage peer-to-peer partnership and movement skills will contribute to achieving these objectives [1].

Gross motor skill activities, such as balancing, crawling, walking, running, cutting and jumping are developed during early childhood and are considered essential elements of motor development [2]. Thus, it seems important to provide preschool children with motor opportunities that favor their motor and cognitive development. Executive functions are a set of cognitive skills necessary for goal-oriented behavior. There is some agreement on considering inhibition and interference control, working memory, and cognitive flexibility as core sub-processes. From core Executive function, higher-order Executive function are built, including planning (choosing steps to reach a goal), reasoning, and problem-solving. Specifically for TeleSSE, all these processes are needed to planification, execution and adjustment of the different motor patterns of SSE [3].

One tool that could contribute greatly to the development of the aforementioned skills is the educational psychomotor intervention, aimed at children between 0 and 8 years old [4]. It could be useful to help the children to develop their motor, personal and social skills which allow them to adapt to different situations in the preschool setting [5]. Many scientific studies support the importance of psychomotor development and providing opportunities to explore the physical environment, manipulate objects and interact with peers in the first years of life, which will ensure a correct impact on their psychomotor development and their interaction with the outside world [6]. Children with greater psychomotor skills and, therefore, greater success in performing physical and psychomotor activities have more social success and popularity among their peers [7]. Furthermore, a relationship has been shown between the opportunities for motor actions available to children during childhood and their physical competence later in life [8]. Several authors also argue that educational psychomotor intervention contributes to the development of motor and personal competences, as well as social and academic skills [5,9]. Specifically, Ruiz-Esteban et al. [10] analyzed the effectiveness of a motor intervention programme on gross motor skills in preschoolers, reporting statistically significant improvements in the development of fundamental motor skills such as leg and arm coordination. Thus, educational psychomotor intervention could be a powerful tool that allows children to better understand their body image, improve their self-concept, learn to communicate, express their ideas and feelings and develop values such as empathy, respect and collaboration, among others [9]. Therefore, there is no doubt that there is a close relationship between educational psychomotor intervention and preschool children’s motor skills [2]. Thus, psychomotor practices and interventions on movement skills that can improve fitness should be considered during the early school years and at the preschool stage [11].

Motor abilities have been widely studied. The relationship between working on motor skills through physical activity has been found in longitudinal studies [12,13,14,15,16,17] and benefits different parameters, such as cardiorespiratory fitness and general health in children [18,19]. One of the methods that has recently been studied is the inclusion of exergaming as a means of physical activity in children [20]. A recent systematic review by McDonough et al. [21] showed the efficacy of using physical activity on motor skill acquisition, also reporting beneficial effects of physical activity promotion. This review also demonstrated that exergaming-based intervention programmes showed a similar relationship to other traditional programmes.

Focusing on exergaming, several studies have shown the benefits on different populations, such as obese children and adolescents [22], autism spectrum disorder [23], cognitive impairment or dementia [24], and even as a means for mitigating fatigue and the negative effects of confinement and quarantine during pandemic situations such as that caused by COVID-19 [25].

A popular modality of exergaming is the square-stepping exercise (SSE), which is an educational psychomotor intervention activity with a playful and innovative character for the development of motor and cognitive skills in preschoolers. It requires both physical and cognitive effort, specifically attention, memory and executive function [26,27]. The SSE consists of the execution of movement patterns including forward, backward, lateral and oblique steps; the complexity of which increases progressively [26]. However, new pattern proposals can be created that include hand steps, jumps, colours, or flexible surfaces, among others, to stimulate children’s gross motor and cognitive skills.

SSE has shown benefits in functionality, balance, lower limb strength, flexibility and agility, concentration, memory, mental acuity and visual memory in older adults [28,29,30]; as well as in preventing and reducing the number of falls, improving cognitive abilities [31]. Additionally, it has been shown to be effective in pathologies such as multiple sclerosis [32] or type 2 diabetes mellitus [33]. However, there is only one study that has analyzed the effects of SSE in preschoolers, showing cognitive and motor benefits in a small sample [34].

Therefore, this project aims to analyze the effects of a 9-month Tele-SSE programme on the motor and cognitive skills in preschool children. More specifically, the present project evaluates the effects of the Tele-SSE-based programme on static balance, physical fitness, body composition, strength, speed-agility, executive function and attention, processing speed, episodic and working memory and language use in preschoolers. In addition, it aims to promote cooperative work among children to foster social skills and interpersonal relationships.

## 2. Material and Methods

### 2.1. Study Design

Due to the nature of the study, the Consolidated Standards of Reporting Trials Statement (CONSORT) methodology [35] will be followed. A randomized controlled trial with a 1 to 1 assignment ratio to experimental and control groups, respectively, will be conducted.

### 2.2. Ethical Approval

The study has been approved by the Bioethics and Biosafety Committee at the University of Extremadura (approval number: 95/2021). The study has been registered in the Clinical Trials Registry provided by the Australian New Zealand Clinical Trial Registry (Request number: 382451; https://www.anzctr.org.au/, accessed on 5 July 2021).

### 2.3. Sample Size

Sample size computations were calculated based on normative values from the Fitness Assessment in the Preschool Battery (PREFIT) for children aged three years old (most heterogeneous population into the selected age range), specifically data from the one-leg balance test [36], because it will be the primary outcome in this study. Thus, accepting a 0.05 alpha risk and a 0.2 beta risk in a bilateral contrast and assuming a common standard deviation of 5.025 and a correlation coefficient of 0.7 between baseline and final measurement [37]; a total of 102 participants (51 subjects in the experimental group and 51 in the control group) are required to detect a difference equal to or greater than 2.43 units. A loss-to-follow-up rate of 20% has been considered.

### 2.4. Randomization and Blinding

Participants will be randomly assigned to experimental (Tele-SSE) or control groups. Research Randomizer computer software (Version 4.0, Geoffrey C. Urbaniak and Scott Plous, Middletown, CT, USA) (http://www.randomizer.org, accessed on 11 June 2021) [38] will be used to create a simple computer-generated randomization sequence before participants are enrolled (1:1). This process will be performed by a research team member without clinical active participation in the trial. The assignment will be hidden in a password-protected computer file. Although participants will know their group assignment, outcome evaluators and data analysts will be blinded to the assignment.

### 2.5. Participants

To be included in the study, participants will have to meet the following eligibility criteria: (a) be children aged between 3 and 6 years old; (b) be enrolled in a public early childhood education centre in the autonomous community of Extremadura; (c) complete and sign the informed consent form by their parents or legal guardians; (d) the children’s consent and voluntariness to participate in the intervention be given; and (e) not be suffering any condition or inconvenience that prevents the normal practice of physical activity.

Furthermore, only participants who complete all evaluations (baseline, quarterly interim, final and follow-up) will be considered for analysis. Likewise, experimental group subjects will need to be involved in at least 80% of the classes. Moreover, it will be necessary to obtain consent and approval by the school council, as well as by the teacher/tutor, to carry out the intervention throughout the school year.

### 2.6. Intervention

#### 2.6.1. Experimental Group

Participants will receive Tele-SSE intervention protocol, three times a week, for nine months. At the beginning of the intervention, an instructor will explain the training guidelines. All sessions will be followed by videoconference through the digital whiteboard placed in every early childhood education classroom. The SSE will be performed on a fine carpet of 200 × 80 cm, divided into 40 squares of 20 × 20 cm, adapted to participants’ morphological characteristics (i.e., squares size will be reduced). Participants will execute the SSE exercises divided in two tapestries simultaneously. The SSE includes a total of 200 different movement patterns which are classified, according to their difficulty, into three general levels: beginner, intermediate and advanced [39]. The beginner level has two sublevels, while the intermediate and advanced levels have 3 sublevels. The proposed intervention will include a progression along the first six levels until the advanced level 1. The greater levels will be combined with other patterns that include colors or flexible surfaces that make the task even more difficult and encourage cooperative work among participants. Likewise, participants will start with simple two-step movement patterns and, step by step, they will perform more complex patterns that will require a greater number of steps per sequence, multidirectional movements and even the performance of other motor skills, such as jumping with feet together, jumping on one leg, etc. Participants should not step on the dividing lines of the squares. Table 1 shows the progression of Tele-SSE training.

Table 2 displays the typical structure that will be followed for Tele-SSE sessions in the experimental group. During the sessions, an avatar will be assigned to each participant, aiming them to register and follow-up their progression, which could encourage the adherence to practice and motivation of participants. Prior to starting every session, the instructor will contact participants by videoconference and will explain to them the session, showing the patterns that are going to be carried out. The first activity, after the relevant general and specific warm-up, will consist of remembering the patterns learnt in previous session. Then, participants will proceed to learn and execute the patterns selected for that day, the number of which will fluctuate between three and five patterns depending on their difficulty. Once the patterns have been performed, children will go on to a cool-down, consisting of stretching and a short relaxation that will help them to return their bodies to their initial state, and move to the next class in a relaxed state. At the end of every session, participants will be asked for their opinion about the patterns they have learned during the session, as well as about the effort they felt after the session using the EPInfant scale [40]. Participants will rate the intensity of the work performed (from 0 to 10) during the cool-down and final reflections, considering the stress and fatigue they have felt during the execution of the session [41,42]. Finally, they will be reminded to go to the bathroom to wash themselves and drink water.

#### 2.6.2. Control Group

Participants will continue with their usual school routines and usual classes, carrying out the scheduled activities (handicrafts, cards, free play, etc.) and will only participate in assessments. Thus, they will not take part in Tele-SSE intervention.

### 2.7. Measures and Procedures

Several measures will be taken to assess the utility and effectiveness of Tele-SSE cognitive and motor program (Table 3).

All measures will be taken using valid and reliable instruments. However, before every assessment session, participants will complete a warm-up where tests procedure will be explained, including a specific familiarization trial before they are carried out. Intersession test reliability will be checked by repeating the tests two weeks after with some of the sample participants. The motor competence evaluation will be carried out applying the PREFIT battery [36], while the assessment of cognitive abilities will be performed using The National Institutes of Health (NIH) Toolbox Early Childhood Cognition Battery (CFB) [43]. The NIH—Early Childhood CFB is part of the NIH Toolbox [44], a comprehensive set of neurobehavioral measurements that quickly assess cognitive, emotional, sensory, and motor functions.

Before starting tests, a warm-up of 3 to 5 min will be carried out, including games consisting of running, jumping and joint mobility exercises. To increase participants’ imagination, motivation, and adherence to the tests, the PREFIT battery uses two fantasy stories that relate the battery tests to play and fun.

On the one hand, the PREFIT battery is the result of a systematic review based on scientific evidence that collects different tests to assess physical fitness in preschoolers. This battery is an efficient tool in terms of time and materials needed and easily applicable to several children at the same time [36]. It includes the following measures:

- Body mass index (BMI). This parameter is calculated as the quotient of participant’s bodyweight (in kilograms) divided by their height (in meters) squared. It will be measured in kg·m^−2^. Bodyweight and height will be measured using a digital scale with measuring rod. Children will be barefoot, standing upright and fully erect, to step onto the centre of the scales distributing their weight between both feet. In addition, they should look straight ahead, place both arms alongside the body and without making any movement to assess their height. Two measurements will be taken, both for bodyweight and height, considering the average of them for analysis.
- Waist circumference. This will be assessed using a non-elastic measuring tape. Children should wear light clothing and stand with their abdomen relaxed and arms crossed over their chest. From this position, the evaluator will encircle the participant’s waist with the tape measure at the level of the navel and parallel to the floor. The participant will then lower his/her arms to a relaxed and abducted position. The measurement will be carried out twice and the average of the two measurements taken for analysis.
- Handgrip strength. This test will be carried out with an analogue dynamometer with an adjustable grip. The children will stand with their elbow extended and without touching their body with the dynamometer, they will slowly and continuously squeeze for at least 2 s, alternatively with both hands. Two attempts will be made with the optimal grip setting and with a short rest between the two attempts. The best attempt from each hand will be taken for analysis. The result of this test will be recorded in kg.
- Lower limb strength. The standing long jump test with feet together will be used. The child will stand behind the jump line on a nonslip surface, with feet shoulder-width apart, flex the knees with arms in front of the body and parallel to the ground, swing arms, push off with force and jump as far as possible. After landing, the subject will remain in a stable position for the attempt to be considered valid. Two attempts will be made and the best of the two will be selected for further analysis. The result of this test will be recorded in cm.
- Speed-agility test. The 4 × 10 m test will be performed on a nonslip surface. For the execution of this test, two parallel lines will be drawn on the ground, 10 m apart from each other and with an evaluator on each line. On the signal of the evaluator, the child will run as fast as possible to the other line, high-five evaluator 2 and return to the initial line to high-five evaluator 1. Then, without stopping, he/she will repeat the same action until completing a total of 4 runs of 10 m each. The time taken by the child to complete the test is measured. The result is recorded in seconds.
- Balance. This will be assessed using the one-leg balance test. Participants will stand still on the ground with one leg flexed. Two attempts will be made with each leg, selecting the best time to hold that position with each leg. Result will be recorded in seconds.
- Cardiorespiratory fitness. The 20-m shuttle run test will be conducted. Children will run between two lines separated by 20 m. Speed will be controlled using an audio signal previously established. The test starts at 8.5 km/h, and the speed will increase by 0.5 km/h every minute. Participants will start at the first audio signal or beep. The test is finished when the participant stops due to fatigue or fails to reach the end line concurrent with the audio signal or beep on two consecutive occasions. The last half stage completed will be considered for analysis.

On the other hand, The NIH—Early Childhood CFB measures cognitive aspects from 3–6 years and includes tests for processes such as executive functions and attention, episodic memory, and language. It is an easy-to-use (through an iPad) and widely applicable test for different populations that includes [43,45,46]:

- Executive function and attention. The two tests within the battery will be performed: the flanker inhibitory control and attention test, a measure of inhibitory control in the context of selective visual attention, and the dimensional change card sort, related to the cognitive flexibility and attention. In the first one, children must focus on a stimulus while inhibiting attention to the flanking ones (fish flanked by two fish on each side). In the second one, two target pictures are presented which vary in two dimensions (shape and colour). Children must sort them as indicated by an audio-recorded cue word. These two tests can be applied to children between 3 and 7 years.
- Episodic memory. This will be assessed with the picture sequence memory test, with different versions for 3–4 and 5–6 years. In any case, children must reproduce previously-shown object sequences.
- Language. This will use the picture vocabulary test, which covers from 3 years and above. The children must select the picture that most closely matches the meaning of an audio-recorded word.

### 2.8. Statistics

Statistical procedures and analysis will be conducted using the Statistical Package for Social Sciences (SPSS, Version 25, IBM SPSS, Armonk, NY, USA) software. Personal data will be kept anonymous.

Kolmogorov−Smirnov and Levene’s test will be used to check the normality and homogeneity of data, respectively. Descriptive data will be expressed as mean and standard deviation (parametric variables) or median and interquartile range (nonparametric variables). Then, independent sample T-test (parametric variables) or Mann−Whitney U-test (nonparametric variables) would be performed to determine whether experimental and control groups are comparable at baseline in terms of participant characteristics. Likewise, to detect whether the cognitive and motor intervention consisting of a one-course (9 months) Tele-SSE program has a statistically significant effect on the motor and cognitive skills of preschoolers, an ANCOVA will be conducted using baseline of the main variable as covariate. Alpha level will be set at *p* ≤ 0.05. The magnitude of differences will be evaluated by Cohen’s d effect size. The effect size thresholds will be interpreted as follows: <0.2, small; 0.2 to <0.8, moderate; ≥0.8, large [47].

## 3. Discussion

The implementation of SSE interventions has been shown to be effective in the older-adult population [28,29,30], as well as in specific populations, such as those with type 2 diabetes mellitus [33], multiple sclerosis [32], Parkinson’s disease [48] or fibromyalgia [39], among others. Thus, we hypothesise that a 9-month intervention based on Tele-SSE will improve motor and cognitive skills in preschoolers. More specifically, Tele-SSE will enhance static balance, physical fitness, body composition, strength, speed-agility, executive function and attention, processing speed, episodic and working memory and language use of preschool children.

This pedagogical proposal will be carried out telematically for several reasons. In addition to questions of motivation and adherence of the participants, as we are living in a pandemic context, as a tool to maintain security in the educational community: it would help to keep the bubble groups intact, and it would be a tool that could help to reduce the contact of teachers who attend several schools and must change location throughout their day, among others. In addition, the implementation of this pedagogical proposal through new technologies allows it to be implemented at any time during the school day and in different places at the same time, as it could be developed synchronously or asynchronously in different preschooler education classrooms in different public schools. Furthermore, its potential applicability to other populations is noteworthy due to its high adherence and viability as a consequence of its originality and its relatively low cost [31].

Regarding early years children, their holistic development is one of the main objectives to be achieved in any educational action or stage [1] and the necessary means and opportunities should be provided to stimulate all their developmental areas: motor, cognitive, emotional and social. To achieve this objective, educational psychomotor intervention should be a powerful tool to contribute to motor, cognitive, social, emotional, and academic skill development [5,9].

This protocol proposes the implementation of a Tele-SEE intervention using digital whiteboards, available in early years educational settings. It is an exergaming modality with great potential for stimulating children’s development, focusing on the motor and cognitive areas. Moreover, Tele-SEE has great potential due to its ease of implementation, the possibility of being used in groups and its wide possibilities of adaptation according to the children’s characteristics.

Therefore, the pedagogical proposal of an educational psychomotor intervention proposed through Tele-SSE would be pioneering, measuring the effectiveness of this type of intervention as a tool for improving motor development, especially balance, and cognitive development in preschoolers between 3 and 6 years. 

## 4. Conclusions

This project will investigate the effectiveness of Tele-SSE in early childhood education children for 9 months, with the aim of improving cognitive and motor skills, especially balance, as well as cognitive skills in preschoolers aged between 3 and 6 years old. If this intervention proves to be effective, it could be implemented in those centers, entities and associations specialized in early childhood education.

## Figures and Tables

**Table 1:** Progression of Tele-SSE intervention proposal.

Month	Frequency (Days a Week)	Session Duration (min)	Steps per Sequence (Number)	Difficulty (Level)	Additional Difficulty Variables
1	3	60	2	Beginner 1	
2	3	60	4	Beginner 1 and 2	
3	3	60	4	Beginner 2	
4	3	60	6	Intermediate 1	
5	3	60	6	Intermediate 2	
6	3	60	6	Intermediate 3	
7	3	60	8	Intermediate 3	Colors
8	3	60	8	Advanced 1	Colors
9	3	60	8	Advanced 1	Flexible surface

**Table 2:** Typical structure of Tele-SSE session.

Typical SSE Session Way
**Warm-up** (10 min)
General
Joint mobility
Stretching
Specific
Cognitive play
Motion game
**Main Part** (40 min)
Review of the patterns learnt in the previous session
Learning and implementation of SSE pattern 1
Learning and implementation of SSE pattern 2
Learning and implementation of SSE pattern 3
Learning and implementation of SSE pattern 4
**Cool-down** (10 min)
Stretching
Relaxation
Final thought
Personal hygiene

**Table 3:** Assessments schedule for both experimental and control group.

Assessment	Baseline	Month 3	Month 6	Month 9	Beginning of Next Course
BMI	x	x	x	x	x
Waist circumference	x	x	x	x	x
Handgrip strength	x	x	x	x	x
Lower limb strength	x	x	x	x	x
Speed-agility	x	x	x	x	x
Balance	x	x	x	x	x
Cardiorespiratory fitness	x	x	x	x	x
Executive function and attention	x	x	x	x	x
Episodic memory	x	x	x	x	x
Language	x	x	x	x	x

BMI, body mass index; x, measure to be taken on that period of time.

## Data Availability

Not applicable.

## References

[1] Educación C.D. (2008). Decreto 4/2008, de 11 de Enero, Por el que se Aprueba el Currículo de Educación Infantil Para la Comunidad Autónoma de Extremadura.

[2] Mostafavi R., Ziaee V., Akbari H., Haji-Hosseini S. (2013). The effects of spark physical education program on fundamental motor skills in 4–6 year-old children. Iran. J. Pediatr..

[3] Diamond A., Bialystok E., Craik F.I.M. (2006). The early development of executive functions. Lifespan Cognition: Mechanisms of Change.

[4] Vecchiato M. (2003). Terapia Psicomotora.

[5] Pons Rodríguez R., Arufe-Giráldez V. (2016). Análisis descriptivo de las sesiones e instalaciones de psicomotricidad en el aula de Educación Infantil. Sportis.

[6] Moreira M.S., Almeida G.N., Marinho S.M. (2016). Efectos de un programa de Psicomotricidad Educativa en niños en edad preescolar. Sport. Rev. Técnico-Científica Deporte Esc. Educ. Física Psicomot..

[7] Evans J., Roberts G.C. (1987). Physical competence and the development of children’s peer relations. Quest.

[8] Brewer C. (2011). Physical and movement skill development. Coaching Children in Sport.

[9] de Aquino M.F.S., Browne R.A.V., Sales M.M., Dantas R.A.E. (2012). A psicomotricidade como ferramenta da educação física na educação infantil. RBFF-Rev. Bras. Futsal Futeb..

[10] Ruiz-Esteban C., Terry Andrés J., Méndez I., Morales Á. (2020). Analysis of motor intervention program on the development of gross motor skills in preschoolers. Int. J. Environ. Res. Public Health.

[11] Hardy L.L., Reinten-Reynolds T., Espinel P., Zask A., Okely A.D. (2012). Prevalence and correlates of low fundamental movement skill competency in children. Pediatrics.

[12] Fisher A., Reilly J.J., Kelly L.A., Montgomery C., Williamson A., Paton J.Y., Grant S. (2005). Fundamental movement skills and habitual physical activity in young children. Med. Sci. Sports Exerc..

[13] Iivonen K., Sääkslahti A., Mehtälä A., Villberg J., Tammelin T., Kulmala J., Poskiparta M. (2013). Relationship between fundamental motor skills and physical activity in 4-year-old preschool children. Percept. Mot. Ski..

[14] Mazzoli E., Koorts H., Salmon J., Pesce C., May T., Teo W.-P., Barnett L.M. (2019). Feasibility of breaking up sitting time in mainstream and special schools with a cognitively challenging motor task. J. Sport Health Sci..

[15] Palmer K.K., Chinn K.M., Robinson L.E. (2019). The effect of the CHAMP intervention on fundamental motor skills and outdoor physical activity in preschoolers. J. Sport Health Sci..

[16] Webster E.K., Martin C.K., Staiano A.E. (2019). Fundamental motor skills, screen-time, and physical activity in preschoolers. J. Sport Health Sci..

[17] Williams H.G., Pfeiffer K.A., O’neill J.R., Dowda M., McIver K.L., Brown W.H., Pate R.R. (2008). Motor skill performance and physical activity in preschool children. Obesity.

[18] Barnett L.M., Van Beurden E., Morgan P.J., Brooks L.O., Zask A., Beard J.R. (2009). Six year follow-up of students who participated in a school-based physical activity intervention: A longitudinal cohort study. Int. J. Behav. Nutr. Phys. Act..

[19] Hands B., Larkin D., Parker H., Straker L., Perry M. (2009). The relationship among physical activity, motor competence and health-related fitness in 14-year-old adolescents. Scand. J. Med. Sci. Sports.

[20] Bonnechère B., Jansen B., Omelina L., Degelaen M., Wermenbol V., Rooze M., Jan S.V.S. (2014). Can serious games be incorporated with conventional treatment of children with cerebral palsy?. A review. Res. Dev. Disabil..

[21] McDonough D.J., Liu W., Gao Z. (2020). Effects of Physical Activity on Children’s Motor Skill Development: A Systematic Review of Randomized Controlled Trials. BioMed Res. Int..

[22] Andrade A., Correia C.K., Coimbra D.R. (2019). The psychological effects of exergames for children and adolescents with obesity: A systematic review and meta-analysis. Cyberpsychology Behav. Soc. Netw..

[23] Ruggeri A., Dancel A., Johnson R., Sargent B. (2020). The effect of motor and physical activity intervention on motor outcomes of children with autism spectrum disorder: A systematic review. Autism.

[24] Zhao Y., Feng H., Wu X., Du Y., Yang X., Hu M., Ning H., Liao L., Chen H., Zhao Y. (2020). Effectiveness of exergaming in improving cognitive and physical function in people with mild cognitive impairment or dementia: Systematic review. JMIR Serious Games.

[25] Chtourou H., Trabelsi K., H’mida C., Boukhris O., Glenn J.M., Brach M., Bentlage E., Bott N., Shephard R.J., Ammar A. (2020). Staying physically active during the quarantine and self-isolation period for controlling and mitigating the COVID-19 pandemic: A systematic overview of the literature. Front. Psychol..

[26] Shigematsu R., Okura T. (2006). A novel exercise for improving lower-extremity functional fitness in the elderly. Aging Clin. Exp. Res..

[27] Varela Álvarez E. (2015). El’Square Stepping Exercise’en Personas Mayores: Una Nueva Forma de Rehabilitación Física y Cognitiva. https://ruc.udc.es/dspace/handle/2183/14592.

[28] Teixeira C.V.L., Gobbi S., Pereira J.R., Ueno D.T., Shigematsu R., Gobbi L.T.B. (2013). Effect of square-stepping exercise and basic exercises on functional fitness of older adults. Geriatr. Gerontol. Int..

[29] Teixeira C.V.L., Gobbi S., Pereira J.R., Vital T.M., Hernandéz S.S.S., Shigematsu R., Gobbi L.T.B. (2013). Effects of square-stepping exercise on cognitive functions of older people. Psychogeriatrics.

[30] Shigematsu R., Okura T., Nakagaichi M., Tanaka K., Sakai T., Kitazumi S., Rantanen T. (2008). Square-stepping exercise and fall risk factors in older adults: A single-blind, randomized controlled trial. J. Gerontol. Ser. A Biol. Sci. Med Sci..

[31] Giannouli E., Morat T., Zijlstra W. (2020). A Novel Square-Stepping Exercise Program for Older Adults (StepIt): Rationale and Implications for Falls Prevention. Front. Med..

[32] Sebastião E., McAuley E., Shigematsu R., Motl R.W. (2017). Feasibility study design and methods for a home-based, square-stepping exercise program among older adults with multiple sclerosis: The SSE-MS project. Contemp. Clin. Trials Commun..

[33] Shellington E.M., Reichert S.M., Heath M., Gill D.P., Shigematsu R., Petrella R.J. (2018). Results from a feasibility study of square-stepping exercise in older adults with type 2 diabetes and self-reported cognitive complaints to improve global cognitive functioning. Can. J. Diabetes.

[34] Ramah N. (2014). The effects of Square-Stepping Exercises on cognitive skills for kindergarten age children. Ovidius Univ. Ann. Ser. Phys. Educ. Sport/Sci. Mov. Health.

[35] Turner L., Shamseer L., Altman D.G., Weeks L., Peters J., Kober T., Dias S., Schulz K.F., Plint A.C., Moher D. (2012). Consolidated standards of reporting trials (CONSORT) and the completeness of reporting of randomised controlled trials (RCTs) published in medical journals. Cochrane Database Syst. Rev..

[36] Cadenas-Sanchez C., Martinez-Tellez B., Sanchez-Delgado G., Mora-Gonzalez J., Castro-Piñero J., Löf M., Ruiz J.R., Ortega F.B. (2016). Assessing physical fitness in preschool children: Feasibility, reliability and practical recommendations for the PREFIT battery. J. Sci. Med. Sport.

[37] Estrada E., Ferrer E., Pardo A. (2019). Statistics for evaluating pre-post change: Relation between change in the distribution center and change in the individual scores. Front. Psychol..

[38] Urbaniak G., Plous S. Research Randomizer (Version 4.0) [Computer Software]. http://www.randomizer.org/.

[39] Carlos-Vivas J., Pérez-Gómez J., Delgado-Gil S., Campos-López J.C., Granado-Sánchez M., Rojo-Ramos J., Muñoz-Bermejo L., Barrios-Fernandez S., Mendoza-Muñoz M., Prado-Solano A. (2020). Cost-Effectiveness of “Tele-Square Step Exercise” for Falls Prevention in Fibromyalgia Patients: A Study Protocol. Int. J. Environ. Res. Public Health.

[40] Rodríguez I., Zenteno D., Cisternas L., Rodríguez P., Reyes G., Troncoso K. (2015). Construcción y evaluación de Epinfant: Una escala para la medición del esfuerzo percibido en la población pediátrica. Arch. Argent. Pediatría.

[41] Borg G. (1990). Psychophysical scaling with applications in physical work and the perception of exertion. Scand. J. Work Environ. Health.

[42] Groslambert A., Mahon A.D. (2006). Perceived exertion. Sports Med..

[43] Weintraub S., Bauer P.J., Zelazo P.D., Wallner-Allen K., Dikmen S.S., Heaton R.K., Tulsky D.S., Slotkin J., Blitz D.L., Carlozzi N.E. (2013). I. NIH Toolbox Cognition Battery (CB): Introduction and pediatric data. Monogr. Soc. Res. Child. Dev..

[44] Gershon R.C., Cella D., Fox N.A., Havlik R.J., Hendrie H.C., Wagster M.V. (2010). Assessment of neurological and behavioural function: The NIH Toolbox. Lancet Neurol..

[45] Casaletto K.B., Umlauf A., Marquine M., Beaumont J.L., Mungas D., Gershon R., Slotkin J., Akshoomoff N., Heaton R.K. (2016). Demographically corrected normative standards for the Spanish language version of the NIH toolbox cognition battery. J. Int. Neuropsychol. Soc..

[46] Fox R.S., Manly J.J., Slotkin J., Devin Peipert J., Gershon R.C. (2021). Reliability and Validity of the Spanish-Language Version of the NIH Toolbox. Assessment.

[47] Cohen J. (1988). Statistical Power Analysis for the Behavioral Sciences.

[48] Mayoral-Moreno A., Chimpén-López C.A., Rodríguez-Santos L., Ramos-Fuentes M.I., Vaz-Leal F.J., Moral M.A., Pérez-Gómez J., Adsuar J.C. (2021). Falls Prevention and Quality of Life Improvement by Square Stepping Exercise in People with Parkinson’s Disease: Project Report. J. Pers. Med..

